# 3D Melt-Extrusion Printing of Medium Chain Length Polyhydroxyalkanoates and Their Application as Antibiotic-Free Antibacterial Scaffolds for Bone Regeneration

**DOI:** 10.1021/acsbiomaterials.4c00624

**Published:** 2024-07-26

**Authors:** Elena Marcello, Rinat Nigmatullin, Pooja Basnett, Muhammad Maqbool, M. Auxiliadora Prieto, Jonathan C. Knowles, Aldo R. Boccaccini, Ipsita Roy

**Affiliations:** † Faculty of Science and Technology, College of Liberal Arts, University of Westminster, London W1W 6UW, U.K.; ‡ Institute of Biomaterials, Department of Materials Science and Engineering, University of Erlangen-Nuremberg, Erlangen 91058, Germany; § Lucideon Ltd., Stoke-on-Trent ST4 7LQ, Staffordshire U.K.; ∥ CAM Bioceramics B.V., Zernikedreef 6, 2333 CL Leiden, The Netherlands; ⊥ Polymer Biotechnology Lab, Centro de Investigaciones Biológicas-Margarita Salas, Spanish National Research Council (CIB-CSIC), Madrid 28040, Spain; # Division of Biomaterials and Tissue Engineering, University College London Eastman Dental Institute, London NW3 2PF, U.K.; ∇ Department of Nanobiomedical Science and BK21 Plus NBM, Global Research Center for Regenerative Medicine, Dankook University, Cheonan 31116, South Korea; ◆ Department of Materials Science and Engineering, Faculty of Engineering, University of Sheffield, Sheffield S3 7HQ, U.K.; ○ Insigneo Institute for In Silico Medicine, University of Sheffield, Sheffield S3 7HQ, U.K.

**Keywords:** polyhydroxyalkanoates, 3D melt-extrusion, hydroxyapatite, antibacterial, bone regeneration

## Abstract

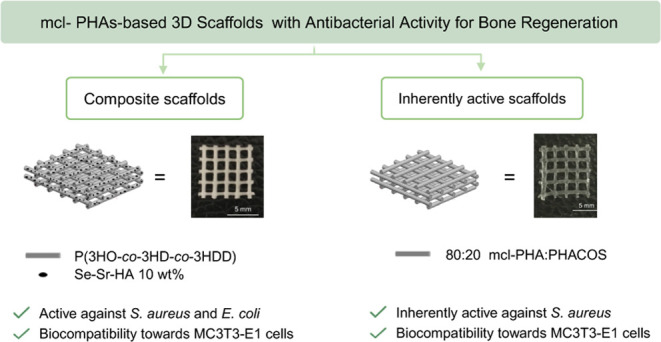

In this work, we investigated, for the first time, the possibility of developing scaffolds for bone tissue engineering through three-dimensional (3D) melt-extrusion printing of medium chain length polyhydroxyalkanoate (mcl-PHA) (i.e., poly(3-hydroxyoctanoate-*co*-hydroxydecanoate-*co*-hydroxydodecanoate), P(3HO-*co*-3HD-*co*-3HDD)). The process parameters were successfully optimized to produce well-defined and reproducible 3D P(3HO-*co*-3HD-*co*-3HDD) scaffolds, showing high cell viability (100%) toward both undifferentiated and differentiated MC3T3-E1 cells. To introduce antibacterial features in the developed scaffolds, two strategies were investigated. For the first strategy, P(3HO-*co*-3HD-*co*-3HDD) was combined with PHAs containing thioester groups in their side chains (i.e., PHACOS), inherently antibacterial PHAs. The 3D blend scaffolds were able to induce a 70% reduction of *Staphylococcus aureus* 6538P cells by direct contact testing, confirming their antibacterial properties. Additionally, the scaffolds were able to support the growth of MC3T3-E1 cells, showing the potential for bone regeneration. For the second strategy, composite materials were produced by the combination of P(3HO-*co*-3HD-*co*-HDD) with a novel antibacterial hydroxyapatite doped with selenium and strontium ions (Se–Sr–HA). The composite material with 10 wt % Se–Sr–HA as a filler showed high antibacterial activity against both Gram-positive (*S. aureus* 6538P) and Gram-negative bacteria (*Escherichia coli* 8739), through a dual mechanism: by direct contact (inducing 80% reduction of both bacterial strains) and through the release of active ions (leading to a 54% bacterial cell count reduction for *S. aureus* 6538P and 30% for *E. coli* 8739 after 24 h). Moreover, the composite scaffolds showed high viability of MC3T3-E1 cells through both indirect and direct testing, showing promising results for their application in bone tissue engineering.

## 1 Introduction

Even though bone has an intrinsic capability of self-healing, it is one of the most transplanted tissues in the world, second only to blood.^1^ Large trauma, tumor resection, or infections can lead to the formation of large-size bone defects or nonunion fractures, requiring the use of additional materials since mechanical fixation alone is not capable of inducing bone repair.^1,2^ Thanks to its inherent osteogenic, osteoconductive, and osteoinductive properties, autologous tissue still represents the gold standard in clinical practice. However, such material has limitations associated with its restricted availability and possible donor site morbidity, especially for the treatment of critical-size defects.^3,4^ In light of such limitations, great attention in the literature has been focused on the development of synthetic bone substitutes, thanks to their unlimited availability and longer shelf life.^1,3^ Recently, a lot of interest has emerged in the development of bone scaffolds with antibacterial properties.^5,6^ In the presence of bacterial bone infection, the regeneration of the tissue is impaired, possibly leading to chronic infection, bone necrosis, and spreading to adjacent tissues. Moreover, resections due to infections represent one of the major causes of large bone size defects. The most common pathogen involved is *Staphylococcus aureus*, but other microorganisms have also been associated with such pathology including *Staphylococcus epidermidis*, *Streptococci sp*., *Pseudomonas sp.*, and *Escherichia coli*.^7,8^ The rise of antibiotic resistance has exacerbated such situation, making antibacterial properties an important requirement for bone regeneration to prevent the rise of infection or to combat existing ones.^9^

Polyhydroxyalkanoates (PHAs) are a family of polyesters produced by bacterial fermentation that have attracted attention in recent years as materials for tissue engineering applications as possible alternatives to synthetic petroleum-derived polymers, thanks to their biocompatibility, biodegradability, easy processability, and versatile and tunable physical and mechanical properties. PHAs can be divided into two classes, short chain length PHAs (scl-PHAs) characterized by a stiff and brittle behavior (with Young’s modulus of 0.5–4 GPa and limited elongation at break <50%), and medium chain length PHAs (mcl-PHAs), elastomeric materials with a high elongation at break (200–500%) and an elastic modulus of 4–50 MPa.

Bone regeneration is one of the main areas of investigation of PHAs in the medical field.^10,11^ The use of both scl and mcl-PHAs has been investigated for load-bearing and nonload-bearing applications, respectively.^12,13^ As PHAs are not inherently osteoconductive, they are primarily investigated in combination with inorganic substrates (e.g., hydroxyapatite or bioglasses) for the development of composite materials.^14^ PHA-based scaffolds for bone regeneration have been mainly produced using conventional processes, such as solvent casting/particulate leaching, electrospinning, and sintering processes.^15−17^ Even though widely investigated, such techniques possess drawbacks and limitations in their applications for the development of prototypes for tissue regeneration due to their lack of reproducibility and control of geometry, porosity, pore structure, and interconnectivity. For these reasons, in recent years, additive manufacturing has attracted great interest in the field of tissue engineering, allowing to produce complex multilayer structures with high control and reproducibility.^18,19^ To date, only a few studies have been conducted on the use of three-dimensional (3D) printing technology with PHAs for the development of bone substitutes.^20−23^ In particular, laser- and extrusion-based technologies have been employed to develop PHA-based 3D structures. Selective laser sintering (SLS) was applied to produce porous structures of cubical form using poly(3-hydroxybutyrate) (P(3HB)) and the surface of the material was coated with osteogenic growth factors by simple physical absorption to improve the bioactivity of the obtained 3D P(3HB) constructs.^20,21^ An alternative technique was investigated to produce composite materials based on poly(3-hydroxybutyrate-*co*-3-hydroxyvalerate) (P(3HB-*co*-3HV)) using SLS. In this study, microspheres of the polymer loaded with calcium phosphate were produced by an oil-in-water emulsion technique and such microparticles were used as the starting powder material for the construction of the 3D printed scaffolds.^22^ The other 3D printing techniques investigated with PHAs are based on extrusion. A 3D printed composite scaffold was produced through solution-based technology by applying compressed air to a mixture of poly(3-hydroxybutyrate-*co*-3-hydroxhexanoate) (P(3HB-*co*-3HHx)) and bioglasses (BG) dissolved in organic solvents. The 3D constructs showed the ability to stimulate bone repair *in vivo* in a rat model.^23^ Alternatively, Yang et al. produced composite scaffolds by melt-extrusion 3D printing of P(3HB-*co*-3HHx) followed by immersion in solution containing BG to obtain surface functionalization for bone tissue engineering applications.^24^ Compared with solution printing, melt extrusion has the advantage of eliminating the possible toxicity related to the use of organic solvents. Commercial scl-PHA-based filaments, based on a blend of PLA and P(3HB-*co*-3HV) with an 88:22 ratio (PLA: P(3HB-*co*-3HV)), are currently available and distributed by ColorFabb (Belfeld, The Netherlands) for fused-deposition modeling (FDM).^25^ Recently, Gregory et al. investigated the printability of this commercial blend filament through FDM, demonstrating its suitability for the development of a wide range of tissue engineering scaffolds (e.g., patches, tooth implants, osteochondral tissue, stents, and nerve conduits).^26^ Finally, Panaksri et al. conducted a preliminary study to investigate the printability of an mcl-PHA (i.e., poly(3-hydroxyhexanoate-*co*-hydroxyoctanoate-*co*-hydroxydecanoate), P(3HHx-*co*-3HO-*co*-HD)) by melt-extrusion 3D printing, identifying the range of parameters suitable for the production of reproducible scaffolds; however, they did not explore printability of any multilayered structures or investigated possible biomedical applications.^27^

With regards to antibacterial features, PHAs are generally not inherently antibacterial. PHAs containing functional moieties in their side chains can be produced directly by bacterial fermentation through the exploitation of their intrinsic metabolic pathways of production.^28^ In the case of *Pseudomonas* species, the incorporation of specific groups can be achieved using the β-oxidation pathway and structurally related carbon sources by selecting substrates containing the desired functional group that can be metabolized by the bacteria. In this way a wide variety of tailored bacterial polyesters have been produced, containing, for example, unsaturated, aromatic, and halogenated groups.^28,29^ Recently, Escapa et al. discovered a new class of PHA-containing thioester groups in their side chains (i.e., PHACOS). This poly(3-hydroxy-acetylthioalkanoate-*co*-3-hydroxyalkanoate), named PHACOS, was obtained using *Pseudomonas putida* KT2442 using a cofeeding experiment with fatty acids (i.e., decanoic acid) as the “good” substrate to support bacterial growth and polymer accumulation supplemented with carbon sources containing thioester groups as the polymer precursor (i.e., 6-acetylthiohexanoic acid).^30^ The PHACOS family of materials was demonstrated to be inherently antibacterial as they showed activity against methicillin-resistant *S. aureus* (MRSA) both *in vitro* and *in vivo*, even though their mechanism of action has still not been discovered.^31^ Moreover, the polymer was able to support the attachment and proliferation of fibroblasts, showing potential for tissue engineering applications.^31,32^ Another strategy to introduce antibacterial properties into PHA-based materials is through the loading of active compounds. In a previous study, antibacterial composite materials based on the combination of an mcl-PHA with novel selenium and strontium cosubstituted hydroxyapatite (Se–Sr–HA) were investigated. The developed composite materials possess high antibacterial activity against *S. aureus* 6538P and *E. coli* 8739 through two mechanisms: direct contact between the bacteria and the surface of materials and the release of active ions at a concentration capable of inhibiting bacterial growth in a prolonged manner, up to 24 h.^33^

The aim of this work was to develop PHA-based antibacterial scaffolds through melt-extrusion 3D printing. We investigated the possibility of melt-extrusion 3D printing of poly(3-hydroxyoctanoate-*co*-hydroxydecanoate-*co*-hydroxydodecanoate), P(3HO-*co*-3HD-*co*-3HDD), an mcl-PHA produced by *Pseudomonas mendocina* CH50 using coconut oil as the carbon source.^34^ A screening study was conducted to evaluate the optimal printing conditions. The *in vitro* degradation of the obtained scaffold was studied by incubation of the sample in phosphate-buffered saline (PBS) for 6 months. Moreover, the possibility of using these materials for bone regeneration was assessed through *in vitro* compatibility and differentiation studies using a murine preosteoblast cell line, MC3T3-E1. In the second part of this work, we investigated the possibility of introducing antibacterial functionalities in the developed 3D P(3HO-*co*-3HD-*co*-3HDD) scaffolds using two strategies. For the first strategy, P(3HO-*co*-3HD-*co*-3HDD) was combined with PHACOS, inherently antibacterial PHAs. For the second strategy, composite materials were produced by the combination of P(3HO-*co*-3HD-*co*-3HDD) with a novel antibacterial hydroxyapatite doped with selenium and strontium ions (Se–Sr–HA). All of the materials were characterized in terms of morphological, chemical, biological, and antibacterial properties.

## 2 Materials and Methods

### 2.1 Bacterial Strains and Culture Conditions

*P. mendocina* CH50 was obtained from the University of Westminster culture collection. *P. putida* KT2442 is part of the Prof Prieto’s Lab strain collection. For the antibacterial characterization, *S. aureus* 6538P and *E. coli* 8739 were purchased from the American Type Culture Collection (ATCC).

### 2.2 PHA Production

P(3HO-*co*-3HD-*co*-3HDD) was produced by *P. mendocina* CH50 using 20 g/L of coconut oil as previously described in ref (34). The fermentation process was carried out in 15 L bioreactors with a 10 L working volume (Applikon Biotechnology, Tewkesbury, U.K.). The polymers were extracted using a two-stage Soxhlet extraction method.^35^

PHACOS were produced by *P. putida* KT2442 as described in ref (30). The fermentation process was carried out in 1 L shaken flasks (200 mL working volume) at 30 °C and 140 rpm for 24 h. The monomer content was 40% of nonfunctionalized monomers (3-hydroxyoctanoate, 3-hydroxydecanoate, and 3-hydroxyhexanoate monomers) and 60% of functionalized monomers (3-hydroxy-6-acetylthiohexanoate and 3-hydroxy-4-acetylthiobutanoate monomers).

### 2.3 Synthesis of Selenium–Strontium–Hydroxyapatite

Selenium–strontium–hydroxyapatite (Se–Sr–HA) was synthesized by the wet precipitation method, using diammonium hydrogen phosphate (NH_4_)_2_HPO_4_, calcium nitrate tetrahydrate Ca(NO_3_)_2_4H_2_O, sodium selenite Na_2_SeO_3_·5H_2_O, and strontium nitrate Sr(NO_3_)_2_·6H_2_O as precursors as described in ref (33). Microsize Se–Sr–HA powders were obtained through milling as described in ref (36).^36^

### 2.4 Melt-Extrusion 3D Printing of PHAs and PHA-Based Composites/Blends

3D printed scaffolds for bone regeneration were developed using the Cellink Inkredible+ 3D bioprinter (Cellink, Goteborg Sweden) with a stainless-steel nozzle with an inner diameter of 0.600 mm. To optimize the melt-extrusion 3D printing of P(3HO-*co*-3HD-*co*-3HDD), the following parameters were considered: the writing speed (0.5, 1, 2, 4 mm/s), the melting temperature (80, 90, 100, 110 °C), and the pressure of extrusion (50, 100, 200 Pa). Structures with a rectilinear pattern (0/90° lay down pattern) with a 1.5 mm distance between the filaments (i.e., pore size) and the deposition of 2 layers of materials were used as a template for the optimization.

Composite 3D printed scaffolds were obtained using P(3HO-*co*-3HD-*co*-3HDD) and Se–Sr–HA. First, 5% w/v composite films containing 10 wt % Se–Sr–HA were prepared as described in ref (33). These films were cut into small pieces (<5 mm) and then used as the starting material for the printing process. Inherently antibacterial melt-extruded 3D printed structures were obtained using P(3HD-*co*-3HD-*co*-3HDD) and PHACOS. First, films in a ratio 80:20 (P(3HD-*co*-3HD-*co*-3HDD):PHACOS) were prepared by dissolving 5% w/v of 80:20 (P(3HD-*co*-3HD-*co*-3HDD): PHACOS) in chloroform. The films were air-dried for 5 weeks at room temperature to allow complete crystallization of the samples.^37^ These films were cut into small pieces (<5 mm) and then used as the starting material for the printing process.

### 2.5 Physicochemical Characterization of 3D Printed Scaffolds

Preliminary characterization of the polymer was carried out using attenuated total reflectance Fourier transform infrared (ATR-FT-IR) spectroscopy, performed in a spectral range of 4000 to 400 cm^–1^ with a resolution 4 cm^–1^ using a PerkinElmer FT-IR spectrometer Spectrum Two. Scanning electron microscopy (SEM) analysis was performed to evaluate the surface topography of the samples using a Jeol 5610LV-SEM Energy-dispersive X-ray (EDX) spectroscopy was used to perform a qualitative elemental analysis of the materials developed. The data were analyzed using the software IncaEDX Energy System. Micro-CT analysis of 3D composite scaffolds of P(3HO-*co*-3HD-*co*-3HDD) containing 10 wt % Se–Sr–HA was performed to investigate the distribution of the filler inside the constructs. Samples of 2–4 mm in length and 1 mm in width were cut using a surgical blade. A series of X-ray images were collected by rotating the samples at 180° at a rotational step of 1° (Joel 5610LV-SEM) and were analyzed using the Bruker Micro-CT CTan and CTVox software.

### 2.6 *In Vitro* Degradation Studies

Degradation studies were conducted on 1 × 1 cm 3D P(3HO-*co*-3HD-*co*-3HDD) scaffolds by incubating the samples in 3 mL of PBS at 37 °C for 1, 2, 3, 4, 5, and 6 months.

The percentage water uptake (%WU), was calculated as
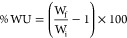
*W*_i_ is the initial hydrated weight and *W*_f_ is the final hydrated weight.

The percentage dried weight variation (%DWV), connected to the solid part loss was calculated as:
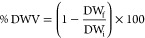
DW_i_ is the initial dried weight and DW_f_ is the final dried weight.

The dried samples were also analyzed with respect to their thermal properties and molecular weight variation. The thermal properties of the samples were analyzed using a differential scanning calorimetry (DSC) instrument, Polyma Netzsch, equipped with an Intracooler IC70 cooling system. One heating ramp cycle (between −70 and 130 °C at 60 °C/min) was performed on each specimen under a nitrogen flow.^37^ The molecular weight of the polymer was determined by gel permeation chromatography (GPC) analysis using a PLgel 5 μm MIXED-C column (Agilent) and analyzed using Agilent GPC/SEC software.

### 2.7 *In Vitro* Cell Biocompatibility Studies

For *in vitro* studies, the MC3T3-E1 subclone 4 (ATCC CRL-2593) mouse cell line bought from ATCC was used. MC3T3-E1 mouse cell line was cultured in α minimum essential medium (α-MEM) with 10% (w/v) fetal bovine serum (FBS) and 1% (w/v) penicillin/streptomycin solution.

#### 2.7.1 Indirect Cytotoxicity

Indirect cytotoxicity of all the produced materials was investigated following ISO 10993-5 procedure for the biological evaluation of medical devices. 5 × 5 mm samples were incubated with 200 μL of α-MEM for 24 h at 37 °C in an atmosphere of 5% CO_2_. 30 000 cells/well were seeded in a 96-well plate and incubated overnight. The following day, the media was removed and replaced with the eluates obtained from the materials. After 24 h, cell viability was calculated using Alamar blue solution, according to the manufacturer’s instructions and compared to that of the control (cells cultivated in the same conditions with nonconditioned medium).

#### 2.7.2 Direct Cytocompatibility

Quantitative evaluation of the cell viability after direct seeding on the surface of the materials was conducted following an adapted protocol described in (34). 5 × 5 mm samples were incubated in 200 μL of α-MEM media for 24 h. After the incubation time, each sample was seeded with a cell seeding density of 70 000 cells/cm^2^ and incubated for 1, 3, and 7 days at 37 °C in an atmosphere of 5% CO_2_. After each time point, Alamar blue assay was performed to evaluate cell viability. The controls consist of cells cultivated on tissue culture plastic for the same time points. Live/dead viability assay was performed on the samples of the direct studies, cultured for 7 days using Calcein AM and Ethidium homodimer-1 (ThermoFisher Scientific LIVE/DEAD Viability/Cytotoxicity Kit Protocol). The samples were imaged by using a confocal microscope (Leica TCS SP2).

#### 2.7.3 *In Vitro* Cell Differentiation Studies

Differentiation studies of MC3T3-E1 cells were conducted on the 3D P(3HO-*co*-3HD-*co*-3HDD) scaffolds. Samples of 5 × 5 mm were cut using a surgical blade and incubated in α-MEM medium for 24 h. After the incubation time, each sample was seeded with a cell seeding density of 70 000 cells/cm^2^ and incubated with α-MEM medium for 24 h. The media was then removed and replaced with 200 μL of osteogenic media (α-MEM complete medium supplemented with 50 μg/mL of ascorbic acid and 10 mM β-glycerophosphate).^100^ The samples were then incubated for 1, 7, 14, and 21 days. At each time point, cell viability was tested using Alamar blue solution.

After cell viability evaluation, the samples were fixed using 200 μL of 4% formaldehyde solution for 30 min. To lyse the cells, 3 freeze-thaw lysis cycles were employed using 0.05% Triton X solution. The supernatant was then used for DNA and alkaline phosphatase (ALP) quantification. The quantification of dsDNA was performed by using the PicoGreen Assay (ThermoFisher Scientific). The ALP activity of MC3T3-E1 cells seeded on the scaffolds was determined as described in refs (38,39). 10 μL of the cell lysate were mixed with 90 μL of 3.6 mM *p*-nitrophenyl phosphate (*p*NPP) substrate solution in 0.5% alkaline buffer solution. The samples were incubated for 1 h at 37 °C. After incubation, 100 μL of 1 M NaOH were added to each well to terminate the reaction. The absorbance of the solution was read at 410 nm using a Spectrostar Nano Microplate Reader (Bmg Labtech, Germany). The ALP activity of each sample was calculated using a calibration curve obtained with *p*-nitrophenol standards. The final ALP activity of cells in nanomoles was normalized to the total content of DNA (ng) present in each sample. The deposition of calcium was evaluated through Alizarin red S (ARS) staining of scaffolds seeded with MC3T3-E1 cells following an adapted protocol by Stanford et al. and Lu et al.^40,41^ After fixing the samples with 200 μL of 4% formaldehyde solution for 30 min, 150 μL of 50 mM ARS solution were added on each scaffold for 20 min at 37 °C. To quantify the calcium deposition, 150 μL of 10% w/v cetylpyridinium chloride in 10 mM sodium phosphate solution were added to each sample and incubated for 1 h at 37 °C. The absorbance was read at 540 nm using a Spectrostar Nano Microplate Reader (Bmg Labtech, Germany).

### 2.8 Antibacterial Characterization

#### 2.8.1 Direct Contact Test—ISO 22196

The antibacterial properties of the 3D composite and inherently antibacterial scaffold were evaluated following ISO 22196 against *S. aureus* 6538P and *E. coli* 8739. 5 × 5 mm samples were placed onto agar plates, inoculated with 20 μL of a bacterial culture adjusted to a concentration of 3–5 × 10^5^ CFU/mL, and incubated in static conditions at 37 °C for 24 h. After the incubation time, the bacteria were recovered from the samples using a PBS solution and the number of viable cells was determined through the colony forming unit method using the drop plate technique.^42^ 3D P(3HO-*co*-3HD-*co*-3HDD) neat samples were used as controls. The antibacterial activity (%bacterial cell reduction) was expressed as the % reduction of the number of cells, which was calculated using the formula
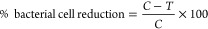
Where *C* is the number of viable bacteria normalized to the surface area of the sample, in CFU/cm^2^, recovered from the control samples, and *T* is the number of viable bacteria, in CFU/cm^2^, recovered from samples containing Se–Sr–HA or PHACOS.

#### 2.8.2 Antibacterial Ion Release Studies

Antibacterial ion release studies were performed on 3D composite antibacterial scaffolds against *S. aureus* 6538P or *E. coli* 8739, following an adapted method described in ref (43). 5 × 5 mm samples were incubated in 1 mL of Mueller Hinton broth at 37 °C for 1, 3, 6, and 24 h. After each time point, the media with the eluted ions were collected and replaced with fresh media. The obtained eluates were incubated with a microbial suspension adjusted to 3–5 × 10^5^ CFU/mL and incubated for 24 h at 37 °C in 96 multiwell plates (final volume 100 μL). The control consisted of MH broth incubated for the same time period with 3D P(3HO-*co*-3HD-*co*-3HDD) neat samples. The antibacterial activity was calculated as the % reduction of the OD at 600 nm compared to the control using the following formula
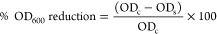


OD_c_ is the optical density at 600 nm of the control samples and OD_s_ is the optical density at 600 nm of the sample.

### 2.9 Statistical Analysis

All measurements were made in triplicate unless otherwise specified, and data are presented as mean values ± standard deviation. Statistical analysis was performed using GraphPad Prism 7 software by One-way analysis of variance (ANOVA) with Tukey posthoc test to determine statistically significant differences between two or more groups. The differences were considered statistically significant when the p-values were lower than 0.05 (*p*-value <0.05 (*), *p*-value <0.01 (**), *p*-value <0.001 (***), and *p*-value <0.0001 (****)).

## 3 Results

### 3.1 Melt-Extrusion 3D Printing of P(3HO-*co*-3HD-*co*-3HDD)

To optimize the melt-extrusion 3D printing of P(3HO-*co*-3HD-*co*-3HDD), the following parameters were considered: the writing speed (i.e., movement of the printer head), the melting temperature, and the pressure of extrusion. Structures with a rectilinear pattern with a 1.5 mm distance between the filaments (i.e., pore size) and the deposition of 2 layers of materials were used as a template for the investigation of such conditions, using a nozzle with an internal diameter of 600 μm. Overall, a combination of the lowest feasible pressure and the maximum feasible writing speed that resulted in reproducible and well-formed scaffolds was considered optimal from an efficiency point of view, as it reduces the energy consumption and the fabrication time.^44,45^ The applied temperature needs to be higher than the melting temperature of the polymer used, but not too high to avoid polymer degradation.^44,45^ Moreover, the lowest temperature achievable is preferred, as it would allow a more rapid solidification of the material. For P(3HO-*co*-3HD-*co*-3HDD), a minimal temperature of 80 °C was required to achieve material extrusion, higher than its melting range between 40 and 60 °C.^34^ The optimal conditions to obtain high reproducibility and shape fidelity were a writing temperature of 110 °C, a pressure of 100 kPa, and a writing speed of 1 mm/s.

**Figure 1:**
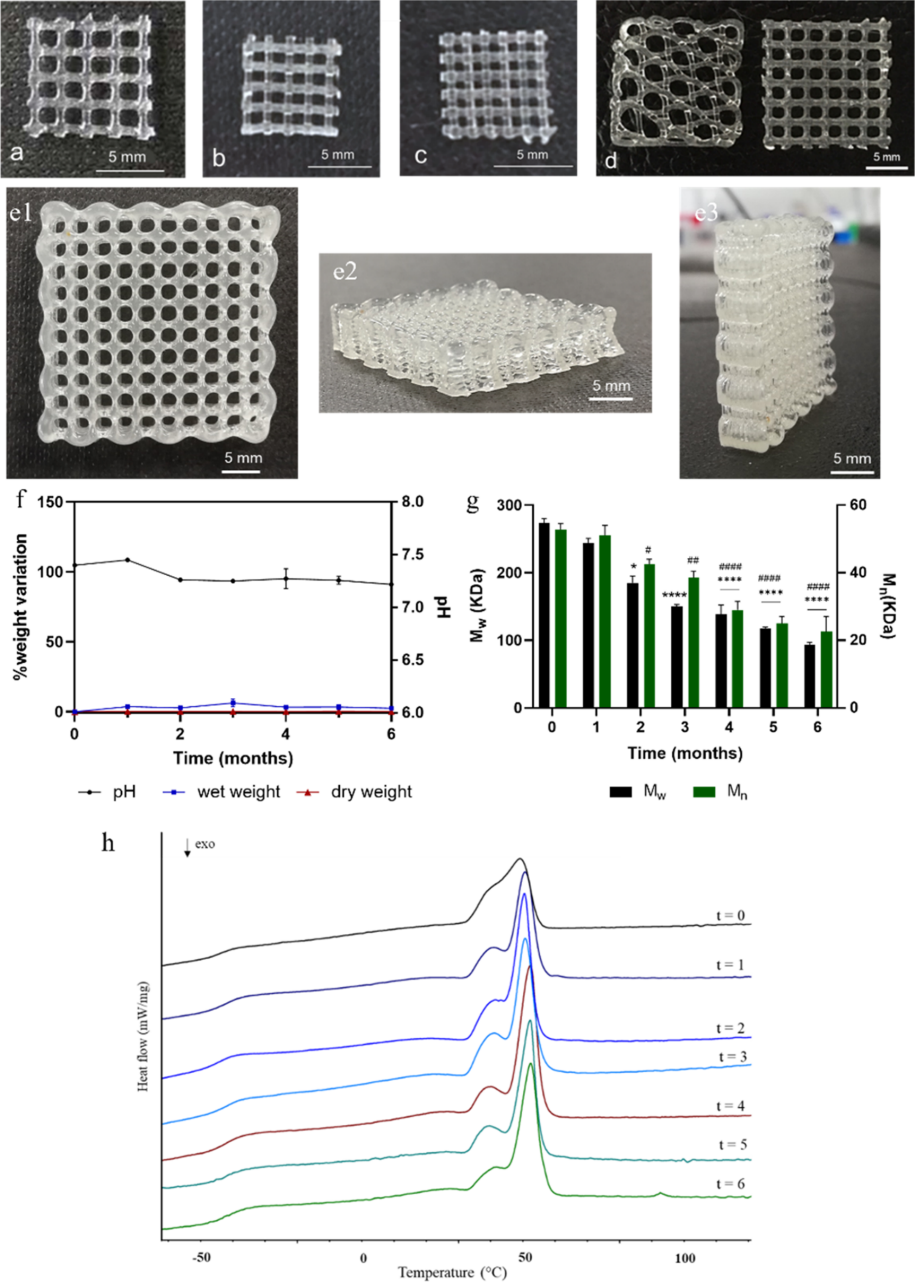
(a–d) 3D printed scaffolds of P(3HO-*co*-3HD-*co*-3HDD) with macro porosities of 1.5 mm × 1.5 mm (A), 1 mm × 1 mm (b), and 0.5 mm × 0.5 mm pore size (c), with different patterns (d), elliptic (left image) and rectilinear (right image), and (e1–e3) with 6 mm of height (1.5 mm × 1.5 mm pore size) obtained by deposition of 22 layers of material. (f) Water uptake, dried weight variation of 3D P(3HO-*co*-3HD-*co*-3HDD) printed scaffolds during 6-month incubation in PBS at 37 °C (*n* = 3), and pH variation in PBS in contact with printed scaffolds. (g) Variation of *M*_w_ and *M*_n_ of 3D P(3HO-*co*-3HD-*co*-3HDD) printed scaffolds during 6-month incubation in PBS at 37 °C (*n* = 3). *, **** indicate statistically significant differences in Mw between the samples at time 0 and all the other time points (*p-*value *<*0.05 and *p-*value *<*0.0001). ^#^, ^##^, and ^####^ indicate statistically significant difference in *M*_n_ between the samples at time 0 and all the other time points (*p-*value *<*0.05, *p-*value *<*0.01, *p-*value *<*0.0001). (h) Representative DSC thermographs for the 3D P(3HO-*co*-3HD-*co*-3HDD) scaffolds during the degradation studies in PBS at 37 °C for 6 months. Thermograms were shifted vertically for better visibility.

Using the optimized conditions, structures with different porosities ranging from 1.5 mm × 1.5 mm to 0.5 mm × 0.5 mm could be obtained, as shown in Figure 1a–c. Moreover, constructs with an elliptic pattern could be obtained, as shown in Figure 1d. All the scaffolds produced possessed a well-defined structure, closely matching that of the implemented design, confirming the printability of the material. Figure 1e1–e3 shows 3D scaffolds of 6 mm height (1.5 × 1.5 mm of pore size). However, the structures obtained did not show porosity in the *z*-axes, an indication of sagging of the material between layers.^46^

#### 3.1.1 Degradation Study

The *in vitro* degradation of the 3D printed structures was investigated by incubating the samples in PBS at 37 °C for 6 months. The materials maintained their shape throughout the course of the study. As shown in Figure 1f, the wet weight of 3D printed structures remained constant throughout the course of the experiments, without statistically significant differences. No statistically significant difference was detected for the % dried weight variation of all the samples analyzed, indicating that the weight remained constant over time. Moreover, no change in pH was observed after 1 month of incubation of the 3D printed scaffolds. After 2 months, a slight but statistically significant decrease in the pH was detected from 7.4 to 7.2 (*p*-value <0.05), and this value remained constant for the rest of the duration of the degradation tests.

The variation of the molecular weights of the samples over time was investigated through GPC analyses. Table 1 and Figure 1g show the weight-average molar weight (*M*_w_), the number-average molar weight (*M*_n_) and the dispersity index (*Đ*_M_ = *M*_w_/*M*_n_) for all the time points evaluated. Overall, the *M*_w_ decreased significantly over time as compared to the sample at time zero, reaching an average reduction in the *M*_w_ by 66% after 6 months of incubation (*p*-value <0.0001). A significant decrease in the average value of *Đ*_M_ was detected after 2 and 3 months of incubation as compared to the initial samples (*p-*value *<*0.05). No statistically significant difference was detected between the other time points evaluated and the initial conditions.

**Table 1:** Variations of the Molecular Weight of 3D P(3HO-*co*-3HD-*co*-3HDD) Printed Scaffolds and Thermal Properties during the Degradation Studies in PBS over 6 Months of Incubation at 37° C^a^

				*T*_m_ (°C)			
time	*M*_w_ (kDa)	*M*_n_ (kDa)	*Đ*_M_	*T*_1_	*T*_2_	*T*_g_ (°C)	Δ*H*_m_ (J/g)
CTR time 0	274 ± 6	52.7 ± 1.8	5.1 ± 0.3	n.d.	50.5 ± 0.5	–43 ± 1.8	17.5 ± 0.5
month 1	244 ± 7	51 ± 3	4.8 ± 0.2	39 ± 0.5	50.5 ± 0.5	–42 ± 0.5	16.7 ± 0.5
month 2	185 ± 10	42.5 ± 1.5	4.3 ± 0.1	39.1 ± 0.3	50.5 ± 0.6	–43 ± 1.2	22.6 ± 1.4
month 3	150 ± 3	38.5 ± 2	3.9 ± 0.2	40 ± 1	51 ± 0.5	–42 ± 0.5	21.2 ± 1
month 4	138 ± 14	29 ± 2.5	4.6 ± 0.3	38.7 ± 1	52.3 ± 0.5	–42.6 ± 2	21 ± 1.9
month 5	117.5 ± 2	25 ± 2	4.6 ± 0.1	38.5 ± 0.5	52.3 ± 0.6	–42 ± 0.5	18.4 ± 1
month 6	93.5 ± 3.5	22.5 ± 4.5	4.3 ± 1	38.5 ± 1	51.7 ± 1.4	–42.6 ± 1.2	19 ± 0.5

a *M*_w_ is the weight-average molecular weight, *M*_n_ is the number-average molecular weight, *Đ*_M_ is the polydispersity index, *T*_m_ is the melting peak (*T*_1_ and *T*_2_ are the two melting peaks evaluated), *T*_g_ is the glass transition temperature and Δ*H*_m_ is the enthalpy of fusion. The results are expressed as average ± standard deviation (*n* = 3). n.d. = not detected.

The variation of the thermal properties during the *in vitro* degradation studies over 6 months are reported in Table 1 and Figure 1h. No statistically significant differences in the glass transition temperature could be detected between the samples during the study. After one month of incubation, a distinct bimodal pattern was detected for the melting peak, characterized by the presence of two melting peaks as shown in Figure 1h. The main peak was at around 51 °C, while the second was at around 39 °C, and the values remained constant over time for all the samples evaluated, without statistically significant differences.

Variations in the enthalpy of fusion of the materials could be detected during the duration of the experiment. No statistically significant differences were evidenced after 1 month of incubation. The enthalpy of fusion increased significantly after 2, 3, and 4 months as compared to the starting material (*p*-value <0.001 for month 3, *p*-value <0.01 for both months 3 and 4). However, no statistically significant differences were evidenced between these time points (i.e., month 2 vs month 3, month 3 vs month 4, and month 2 vs month 4). Finally, no statistically significant differences were detected between the enthalpy values after 5 and 6 months as compared to the initial values.

### 3.2 Biological Characterization

#### 3.2.1 *In Vitro* Cell Compatibility Studies

**Figure 2:**
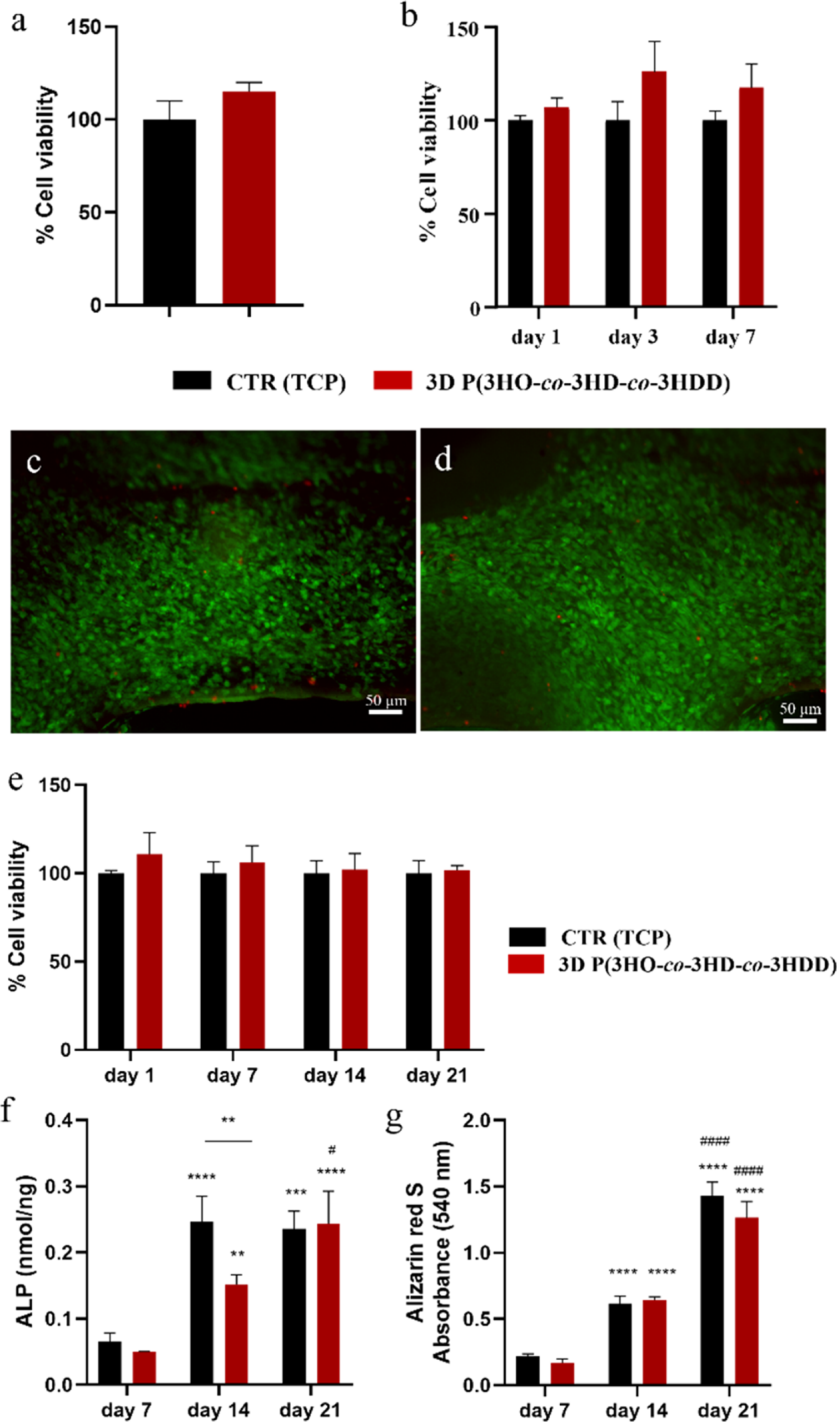
(a) Indirect cytotoxicity studies of 3D P(3HO-*co*-3HD-*co*-3HDD) using the MC3T3-E1 cell line. (b) Cell viability studies of MC3T3-E1 cultured directly on 3D P(3HO-*co*-3HD-*co*-3HDD) at days 1, 3, and 7. Values are expressed as % positive control, i.e., cells cultured directly on tissue culture plastic (TCP) (*n* = 3, error bars = ± SD). There was no statistically significant differences between the samples. (c, d) Representative images of live–dead analysis of the MC3T3–E1 cells cultured on the 3D P(3HO-*co*-3HD-*co*-3HDD) scaffolds for 7 days. Specifically, two sections of the grid-shaped scaffold were reported: (c) the filament part and (d) the “cross shape” central part. (e) Cell viability studies of MC3T3-E1 cells cultured directly on 3D P(3HO-*co*-3HD-*co*-3HDD) at 1, 7, 14, and 21 days cultured using osteogenic media (*n* = 3). (f) Alkaline phosphatase activity of MC3T3-E1 cells cultured on 3D P(3HO-*co*-3HD-*co*-3HDD) using osteogenic media after 7, 14, and 21 days (*n* = 3). The ALP activity was normalized to the DNA content and expressed as nmol/ng. (g) Alizarin red S staining of MC3T3-E1 culture on 3D P(3HO-*co*-3HD-*co*-3HDD) using osteogenic media after 7 and 21 days (*n* = 3). The positive control was TCP. **, ***, **** indicate statistically significant differences between the same samples at day 14 and 21 compared to day 7 (*p-*value *<*0.01, *p-*value *<*0.001, *p-*value *<*0.0001, respectively). ^#^, ^####^ indicate statistically significant difference between the same samples at day 21 compared to day 14 (*p-*value *<*0.05, *p-*value *<*0.0001 respectively).

*In vitro* preliminary cell compatibility studies were conducted to assess the compatibility of the 3D printed scaffolds by using the MC3T3-E1 cell line. Indirect cytotoxicity studies were performed to evaluate the potential release of toxic material from the scaffolds. As shown in Figure 2a, 3D P(3HO-*co*-3HD-*co*-3HDD) printed scaffolds showed cell viability comparable to that of the positive control. This result indicated the absence of toxic degradation products potentially released from the structures developed using melt-extrusion 3D printing. Cell viability studies were conducted to investigate the growth of MC3T3-E1 on the produced materials at 1, 3, and 7 days. As shown in Figure 2b, the cell viability was comparable to that of the positive control (tissue culture plastic, TCP) for all the time points evaluated. A live and dead test was conducted after 7 days of incubation of the MC3T3-E1 cells on the 3D scaffolds to confirm the cell viability results of the Alamar blue assay and evaluate the distribution of the cells on the surface of the materials. Figure 2c,d shows a uniform layer of live cells on the 3D scaffolds, confirming the capability of the scaffold to favor the attachment and proliferation of the cells.

#### 3.2.2 *In Vitro* Cell Differentiation Studies

To further investigate the possibility of using the 3D P(3HO-*co*-3HD-*co*-3HDD) scaffolds for bone tissue engineering, differentiation studies were conducted by culturing MC3T3-E1 cells in osteogenic media containing ascorbic acid and β-glycerophosphate. The cells were seeded on the surface of the materials and incubated for 21 days. Preliminary investigation of the cell viability was conducted using Alamar blue assay, and the results are shown in Figure 2e. The cell viability was comparable with the control (i.e., cell cultured on tissue culture plastic) for all the time points evaluated without statistically significant differences.

To evaluate the differentiation of MC3T3-E1 cells into osteoblasts, the expression of the alkaline phosphatase (ALP) was investigated. This enzyme can hydrolyze phosphate esters bonds and has been shown to promote mineralization by increasing the concentration of inorganic phosphates through the hydrolysis of pyrophosphate.^42^Figure 2f shows the ALP activity for the MC3T3-E1 cells cultured on the 3D P(3HO-*co*-3HD-*co*-3HDD) scaffolds at 7, 14, and 21 days. The average values of ALP activity increased over time for the 3D constructs with a statistically significant difference at both day 14 and day 21 as compared to day 7 (*p*-value <0.02 for day 14 and *p*-value <0.0001 for day 21). At day 7 no statistically significant differences were evidenced between the sample and the positive control. A statically lower average was detected at day 14 for the cells cultured on the scaffolds as compared to the control (*p*-value <0.01). However, the ALP activity of the scaffolds increased between day 14 and day 21 (*p-*value <0.05) reaching the same values as the positive control. No statistically significant difference was in fact observed between the 3D constructs and the TCP after 21 days of incubation.

The Alizarin red S staining solution was used to evaluate the mineralization of the scaffolds at 7, 14, and 21 days. The dye is able to bind with the calcium deposited by a chelation mechanism, allowing the formation of a red complex, which can then be extracted and analyzed by colorimetric detection at 540 nm.^43^Figure 2g shows the results of the Alizarin red S staining for the scaffolds compared to the control. A significant increase in the absorbance was detected for both the scaffold and the positive control from day 7 to day 14 and from day 14 to day 21 (*p*-value <0.0001). No statistically significant difference was evidenced between the 3D printed scaffolds and the positive control for all the three time points considered.

### 3.3 Antibacterial P(3HO-*co*-3HD-*co*-3HDD)-Based Scaffolds for Potential Application in Bone Regeneration

Two strategies were investigated to introduce antibacterial functionalities into the developed 3D P(3HO-*co*-3HD-*co*-3HDD) scaffolds. For the first strategy, inherently antibacterial PHA blends were obtained by combining P(3HO-*co*-3HD-*co*-3HDD) with PHACOS. For the second strategy, composite materials were produced through the combination of P(3HO-*co*-3HD-*co*-HDD) with a novel antibacterial hydroxyapatite containing selenium and strontium ions (Se–Sr–HA).^33^

#### 3.3.1 Inherently Antibacterial PHA Scaffolds

**Figure 3:**
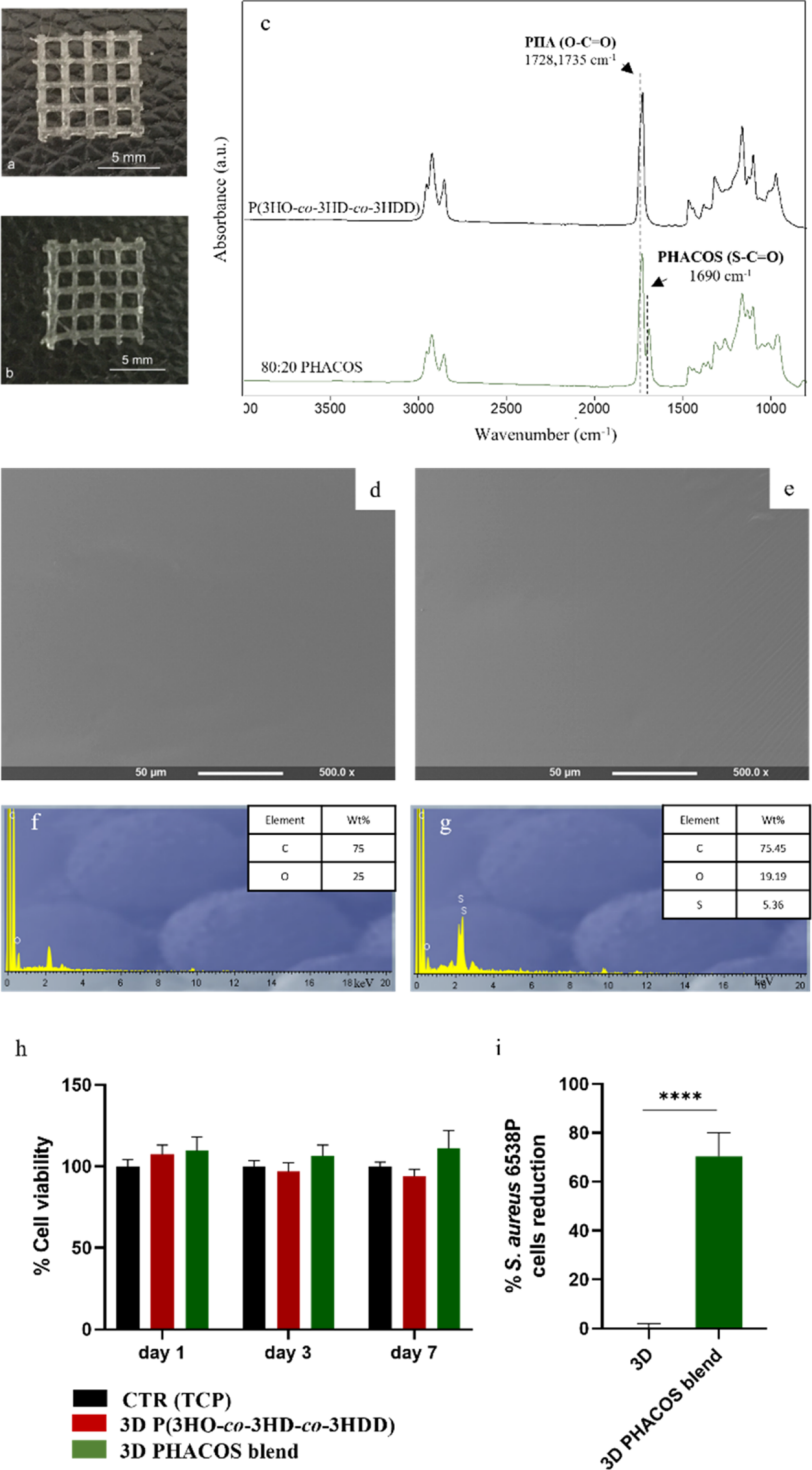
(a, b) Optical image of 3D scaffolds of the P(3HO-*co*-3HD-*co*-3HDD) scaffold (a) and 3D printed 80:20 blend scaffolds of P(3HO-*co*-3HD-*co*-3HDD) and PHACOS (b). (c) ATR-FT-IR spectra of 3D printed scaffolds of P(3HO-*co*-3HD-*co*-3HDD) and 3D printed 80:20 P(3HO-co-3HD-co-3HDD):PHACOS scaffolds. (d, e) SEM images and (f, g) EDX spectra of 3D printed scaffolds of P(3HO-*co*-3HD-*co*-3HDD) (d–f) and 3D printed 80:20 P(3HO-co-3HD-co-3HDD): PHACOS scaffolds (e–g). (h) Cell viability study of MC3T3-E1 cells on the 3D printed 80:20 P(3HO-co-3HD-co-3HDD): PHACOS scaffolds (green) and 3D P(3HO-*co*-3HD-*co*-3HDD) scaffolds (red) at days 1, 3, and 7 (*n* = 3). The positive control was the tissue culture plastic (TCP) (black). There was no statistically significant difference between the samples and the positive control for all time points evaluated (*p*-value >0.5). (i) Antibacterial activity (ISO 22196) against *S. aureus* 6538P evaluated of 3D P(3HO-*co*-3HD-*co*-3HDD) scaffolds and 3D printed 80:20 P(3HO-co-3HD-co-3HDD): PHACOS scaffolds scaffolds (*n* = 6). **** indicates a statistically significant difference between of 3D P(3HO-*co*-3HD-*co*-3HDD) scaffolds and 3D printed 80:20 P(3HO-co-3HD-co-3HDD): PHACOS scaffolds scaffolds (*p-*value *<*0.0001).

To develop inherently antibacterial scaffolds for bone tissue engineering, PHACOS were produced as described in ref (30). PHACOS are completely amorphous in nature and cannot be used as the bulk material for scaffold production. For this reason, PHACOS were blended with P(3HO-*co*-3HD-*co*-3HDD) to produce inherently antibacterial 3D printed constructs. A 80:20 ratio of P(3HO-*co*-3HD-*co*-3HDD) and PHACOS was chosen, as this ratio showed antibacterial activity in blend films (data not shown). As shown in Figure 3b, the 3D printed inherently antibacterial scaffolds could be successfully processed by using melt-extrusion 3D printing. The same printing conditions as those selected for the P(3HO-3HD-3HDD) neat samples (110 °C, 1 mm/s and 100 kPa) were applied for the extrusion of the PHA blend.

#### 3.3.2 Physicochemical Characterization

Preliminary ATR-FT-IR analyses were conducted on the 3D inherently antibacterial scaffolds and are shown in Figure 3c. In the spectra of both materials, the characteristic peaks of PHAs could be detected (i.e., 1720–1740 cm^–1^ related to the stretching of the carbonyl group of the ester bond and around 2900 cm^–1^ related to the stretching of carbon–hydrogen bond of methyl and methylene group (CH_3_, CH_2_)).^47,48^ The spectrum of the 3D blend scaffolds showed an extra peak at 1690 cm^–1^ which is related to the stretching of the carbonyl group present in thioester groups.^49^

The surface morphology of the scaffolds was investigated by SEM analysis. Figure 3e shows that 3D printed inherently antibacterial scaffolds possess a smooth surface without porosity comparable to the 3D printed neat scaffolds (Figure 3d). EDX analysis confirmed the presence of sulfur in the blend scaffolds of PHACOS and P(3HO-*co*-3HD-*co*-3HDD), as shown in Figure 3f,g. The spectra of the neat 3D P(3HO-*co*-3HD-*co*-3HDD) scaffold showed the presence of peaks related to carbon and oxygen, while for 3D printed 80:20 P(3HO-co-3HD-co-3HDD): PHACOS scaffolds new bands related to the sulfur present in the thioester bond were identified.

#### 3.3.3 Biological Characterization

##### 3.3.3.1 *In Vitro* Cell Compatibility Studies

Preliminary *in vitro* cell compatibility studies were conducted using MC3T3-E1 cell line for days 1, 2, and 7. The cell viability was compared to that of the positive control TCP and is reported in Figure 3h. For all the time points considered, the cell viability on the 3D printed 80:20 P(3HO-co-3HD-co-3HDD): PHACOS scaffolds antibacterial scaffolds was comparable with both the tissue culture plastic and 3D P(3HHx-*co*-3HD-*co*-3HD) scaffolds (average cell viability 109% at day 1, 106% at day 3, 111% at day 7).

##### 3.3.3.2 Antibacterial Characterization

The antibacterial activity of the scaffolds was evaluated using ISO 22196 against *S. aureus* 6538P and compared to 3D scaffolds composed only of P(3HO-*co*-3HD-*co*-3HDD). As shown in Figure 3i, the 3D printed blend scaffolds showed antibacterial activity against *S. aureus* 6538P. A decrease in the number of bacterial cells growing on the surface of the material was observed compared with the neat materials. An average 70% reduction in the number of bacterial cells was obtained (*p-*value *<*0.01).

#### 3.3.4 Antibacterial Composite Scaffolds

To develop antibacterial composite scaffolds by melt-extrusion 3D printing, P(3HO-*co*-3HD-*co*-3HDD) composite film developed in a previous work^33^ were used as the starting materials, containing 10 wt% Se-Sr-HA.

**Figure 4:**
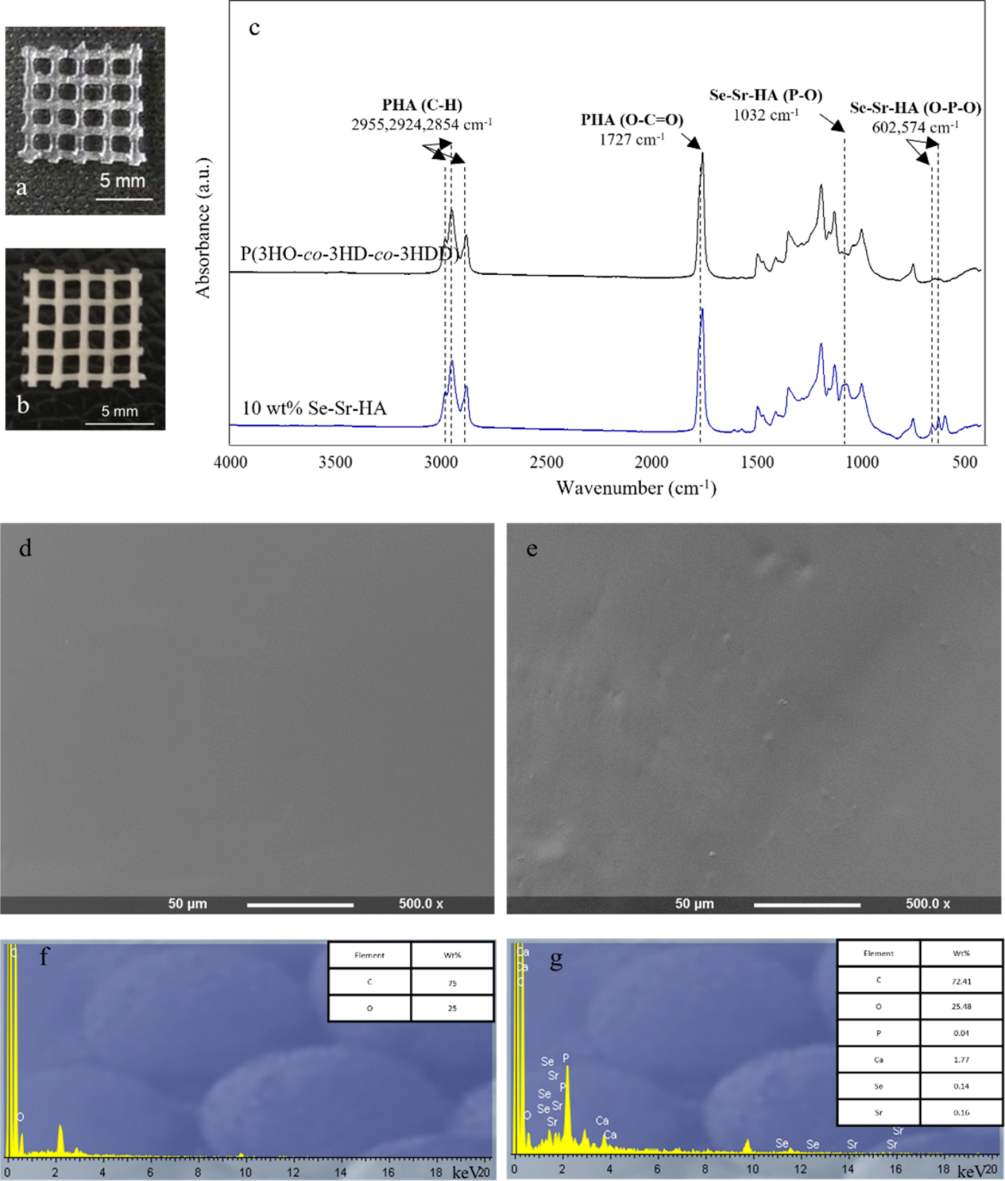
(a, b) Optical image of 3D scaffolds of 3D neat P(3HO-*co*-3HD-*co*-3HDD) scaffold (a) and 3D printed 10 wt % Se–Sr–HA P(3HO-*co*-3HD-*co*-3HDD) composite scaffold (b). (c) ATR-FT-IR spectra of 3D printed scaffolds of P(3HO-*co*-3HD-*co*-3HDD) and 3D printed 10 wt % Se–Sr–HA P(3HO-*co*-3HD-*co*-3HDD) composite scaffolds. (d, e) SEM images and (f, g) EDX spectra of 3D printed scaffolds of P(3HO-*co*-3HD-*co*-3HDD) (d–f) and 3D printed P(3HO-*co*-3HD-*co*-3HDD)/10 wt % Se–Sr–HA composite scaffolds (3-G).

Starting from the optimized printing conditions obtained for 3D melt-extrusion printing of P(3HO-*co*-3HD-*co*-3HDD), a higher temperature of 120 °C and a higher pressure between 170 and 200 kPa were required to extrude the investigated composite composition, as compared to the neat constructs. Visually it was possible to see the presence of hydroxyapatite in the composite scaffolds (Figure 4b). Such materials were characterized by white color and were more opaque than the scaffolds obtained with only P(3HO-*co*-3HD-*co*-3HDD) (Figure 4a).

##### 3.3.4.1 Physicochemical Characterization

Preliminary characterization of the 3D composite structures was conducted through ATR-FT-IR analysis, as shown in Figure 4c. In both spectra, the characteristic peaks of PHAs could be detected. All the spectra showed a peak at around 1720–1740 cm^–1^ related to the stretching of the carbonyl group of the ester bond and around 2900 cm^–1^ related to the stretching of the carbon–hydrogen bond of the methyl and methylene group (CH_3_, CH_2_).^47,48^ In the composite scaffolds, new bands at 1072–1032, 601, 571, and 474 cm^–1^ could be detected compared to the neat 3D constructs. Such peaks were assigned to vibrations of the phosphate group, PO_4_^3–^, present in the Se–Sr–HA.^50^ Moreover, a peak at 630 cm^–1^ could be detected, which was assigned to the vibration of hydroxyl groups (OH) present in hydroxyapatite.^36^

The surface morphology of the scaffolds was investigated by a SEM analysis. Figure 4e shows that the composite materials possessed a smooth surface without the presence of pores, like neat scaffolds (Figure 4d). The presence of Se–Sr–HA could be detected through EDX analyses, as shown in Figure 4g. The spectra of the neat 3D P(3HO-*co*-3HD-*co*-3HDD) scaffold (Figure 4f) showed only the presence of peaks related to carbon and oxygen, while for all the composite constructs new bands related to the characteristic elements of Se–Sr–HA (i.e., calcium, phosphorus, selenium, and strontium) were identified (Figure 4g).

**Figure 5:**
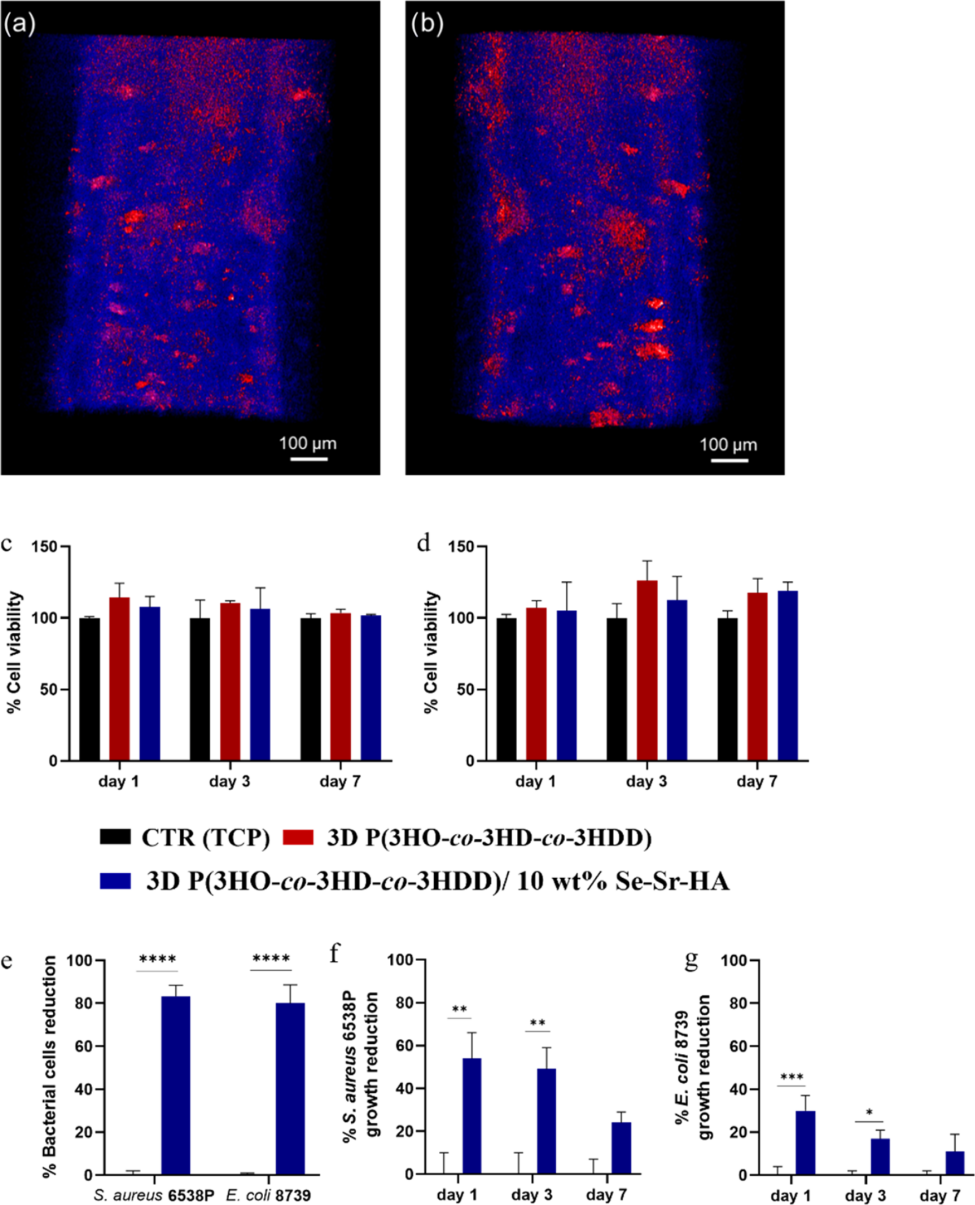
(a, b) Micro-CT reconstructed image of two filaments of 3D P(3HO-*co*-3HD-*co*-3HDD)/10 wt % Se–Sr–HA composite scaffolds. The area in blue represents the polymer, while the area in red represents Se–Sr–HA. (c) Indirect cytotoxicity studies using MC3T3-E1 and (d) direct cell viability study of MC3T3-E1 cells seeded on 3D printed P(3HO-*co*-3HD-*co*-3HDD) neat scaffolds (red) and 3D printed P(3HO-*co*-3HD-*co*-3HDD) antibacterial composite scaffolds with 10 wt % Se–Sr–HA (blue) (*n* = 3). The positive control was tissue culture plastic (TCP)(black). (e) Antibacterial activity (ISO 22196) of 3D melt-extrusion printed P(3HO-*co*-3HD-*co*-3HDD) (red) and 3D printed P(3HO-*co*-3HD-*co*-3HDD) antibacterial composite scaffolds with 10 wt % (blue) *S. aureus* 6538P and *E. coli* 8739 (*n* = 6). (f, g) Indirect antibacterial ion release study of the P(3HO-*co*-3HD-*co*-3HDD) antibacterial composite films with 10 wt % (blue) Se–Sr–HA as a filler against (f) *S. aureus* 6538P and (g) *E. coli* 8739 (*n* = 3). The control consisted of bacterial cells cultured in eluates of 3D neat P(3HO-*co*-3HD-*co*-3HDD) (black). *, **, and *** indicate statistically significant differences between the composite samples and the positive control (*p-*value *<*0.05, *p-*value *<*0.01, *p-*value *<*0.001).

Micro-CT analysis of the 3D P(3HO-*co*-3HD-*co*-3HDD) composite scaffolds containing 10 wt % Se–Sr–HA was performed to qualitatively evaluate the distribution of the filler inside the 3D printed constructs. Figure 5a,b shows the two-dimensional (2D) cross-sectional area of two filaments of 3D P(3HO-*co*-3HD-*co*-3HDD)/10 wt % Se–Sr–HA. The two phases of the composite materials could be distinguished thanks to their difference in absorption of the radiation. Using the Bruker Micro-CT CTan software, two different colors were used to identify the two components present in the composite material. The organic part (i.e., the polymer) led to a lower attenuation and was colored in blue, while higher attenuation was caused by the inorganic phase (i.e., Se–Sr–HA), which was colored in red. Overall, Figure 5a,b shows a uniform distribution of the filler within the 3D printed struts.

##### 3.3.4.2 Biological Characterization

##### 3.3.4.2.1 *In Vitro* Cell Compatibility Studies

The biocompatibility of the 3D printed antibacterial composite scaffolds was tested using MC3T3-E1 cell line to investigate the potential of such materials for bone regeneration.

To investigate the potential release of toxic substances from the materials over time, an indirect cytotoxicity test was conducted on all the composites produced for 1, 3, and 7 days. The cell viability was calculated as a percentage of the positive control (cells cultured on tissue culture plastic) and is shown in Figure 5c. The composite materials with 10 wt % antimicrobial filler showed a cell compatibility comparable to both the positive controls and neat 3D P(3HO-*co*-3HD-*co*-3HDD) samples, without any statistically significant differences.

Direct compatibility tests were performed on all of the composite scaffolds produced. MC3T3-E1 cells were seeded directly on the surface of the materials and cultured for 1, 3, and 7 days. Figure 5d shows the cell viability, which was calculated as a percentage of the positive control (cells cultured on tissue culture plastic). For all the time points evaluated, the cell viability of the 3D composites with 10 wt % Se–Sr–HA was comparable to both the positive control and the 3D neat samples, without statistically significant differences.

##### 3.3.4.2.2 Antibacterial Studies—ISO 22196

The antibacterial properties of the 3D printed composite scaffolds were investigated using the ISO 22196 against *S. aureus* 6538P and *E. coli* 8739. The antibacterial activity was calculated as the reduction in the number of bacterial cells on the composite materials compared to the control. As shown in Figure 5e, the materials were active against both bacterial strains at all of the concentrations tested, and the activity increased with the increase in the hydroxyapatite content. Both compositions showed a similar level of bactericidal effect against the two types of bacteria. Against *S. aureus* 6538P, the 3D printed P(3HO-*co*-3HD-*co*-3HDD)/10 wt % Se–Sr–HA, induced an average of 83% reduction of the number of bacterial cells (*p-*value *<*0.01). A similar behavior was detected for the Gram-negative bacteria, *E. coli* 8739. 3D P(3HO-*co*-3HD-*co*-3HDD)/10 wt % Se–Sr–HA lead to an 80% reduction of the bacterial cell count (*p-*value *<*0.01 compared to 3D neat samples).

##### 3.3.4.2.3 Antibacterial Ion Release Studies

The potential of the material to release ions with antibacterial properties was measured by using an indirect antimicrobial ion release study. The 3D composites containing 10 wt % Se–Sr–HA resulted in a reduction in the optical density of both *S. aureus* 6538P and *E. coli* 8739 cells as compared to the negative control throughout the duration of the experiment as shown in Figure 5f,g. In the case of *S. aureus* 6538P, the materials showed a 54 and 49% reduction in the bacterial cell count at days 1 and 3, respectively, as compared to the control (*p-*value *<*0.01). For day 7, a lower but still significant reduction of the OD could be detected compared to the control, with an average value of 24% reduction in bacterial growth (*p-*value *<*0.05). 3D P(3HO-*co*-3HD-*co*-3HDD)/10 wt % Se–Sr–HA showed activity against *E. coli* 8739, although the effect was lower than that against *S. aureus* 6538P. After 1 day, an average 30% reduction of the OD was obtained as compared to the control (*p-*value *<*0.001). A significant reduction of the bacterial cell count of 17% could be detected with the eluates obtained after incubation for 3 days with the polymeric samples (*p-*value *<*0.05). Finally, the eluates obtained after 7 days of incubation showed an average reduction of bacterial OD of 11%, but no statistically significant difference was detected as compared to the control samples.

## 4 Discussion

In this work, we investigated for the first time the development of mcl-PHA-based scaffolds using melt-extrusion 3D printing to develop biocompatible scaffolds with antibacterial properties for potential applications in bone regeneration. Firstly, the feasibility of developing 3D scaffolds through melt extrusion 3D printing of P(3HO-*co*-3HD-*co*-3HDD) was investigated. To date, only a limited number of studies have been conducted on the 3D printing of PHAs, and only one work investigated the possibility of using an mcl-PHA to produce scaffolds via melt-extrusion 3D printing without evaluating multilayered structures or their possible application for tissue engineering.^21−24,26,27,51^ In this work, a screening study was conducted to evaluate the optimal printing conditions for P(3HO-*co*-3HD-*co*-3HDD). Overall, the screening study allowed the determination of the optimal combination of parameters to successfully print well-defined and reproducible 3D structures using P(3HO-*co*-3HD-*co*-3HDD), for the selected nozzle size (i.e., 600 μm). Future work should be conducted to investigate the effect of the nozzle dimensions on the process. Visual observation of the final 3D melt-extrusion printed scaffolds (i.e., 6 mm height) revealed that no porosity in the *z*-direction could be achieved due to sagging of the material. During the printing process, solidification of each layer must occur before the following layer can be applied to avoid lateral collapse. This feature depends on both the processing parameters (e.g., temperature of printing, the writing speed, the layer height) and the material properties.^52,53^ In this work, variations in operating conditions did not permit the formation of pores in the *z*-direction, indicating a possible intrinsic limitation of the polyester used. Several studies in the literature have indeed shown that PHAs possess low solidification properties from the melt.^54,55^ This mcl-PHA behavior is likely a combined result of very low glass transition temperature and slow crystallization. A possible solution could be explored in the future using dual printing of polyesters with other materials as sacrificial support layers or porogens.^56,57^

Understanding the degradation behavior of the scaffold is essential for biomedical applications, as constructs should be able to degrade in a manner suitable for progressive replacement of the materials over time. Moreover, the degradation product should be nontoxic and should not elicit an adverse reaction at the site of the implantation. *In vivo* degradation is a complex process, depending on both the environmental conditions (e.g., pH, temperature, and presence of cells and enzymes) and the material properties (e.g., polymer chemical structure, molecular weight, crystallinity, morphology).^58,59^ For this reason, preliminary *in vitro* investigations are required to understand the degradation mechanism under simpler conditions.^60^ In this study, the degradation of the 3D printed P(3HO-*co*-3HD-*co*-3HDD) scaffolds was investigated by immersion of the samples in PBS at 37 °C and pH of 7.4 over 6 months.^59,61^ In such conditions, polyesters are known to be degraded by hydrolysis of the ester bonds. In this work, no change in the weight of the 3D scaffold could be detected, while a decrease of the molecular weight (*M*_w_) of up to 66% was observed after six months. In the literature, a few studies have been conducted on the *in vitro* degradation for scl-PHA-based scaffolds, while to date, no study has been conducted on the *in vitro* degradation of mcl-PHAs. Freier et al. showed a reduction of 50% of the molecular weight (*M*_w_) of P(3HB) films after 14 months of incubation, while for P(3HB) electrospun fibers, a 54% decrease of *M*_n_ was observed after 21 months.^58,62^ The degradation of P(3HB-*co*-3HV) fibers has also been investigated showing a similar rate with a 47% decrease of *M*_n_ after 11 months.^63^ These results highlighted that the mcl-PHA investigated in this work showed a faster rate of degradation compared to the scl-PHAs, P(3HB), and P(3HB-*co*-HV) studied in the literature. This behavior could be ascribed to the possible lower crystallinity and highly amorphous nature of P(3HO-*co*-3HD-*co*-3HDD) compared to the scl-PHAs. Crystalline regions are known to reduce the rate of degradation as they limit water penetration due to their tightly packed conformation.^59,64^ The higher susceptibility of the amorphous region compared to the crystalline one could also explain the increase in the enthalpy of fusion (and therefore increase in crystallinity) detected after 2, 3, and 4 months of incubation of the scaffolds. Such a behavior has been evidenced for several polyesters including PHAs and it has been attributed to the fact that the hydrolysis of the amorphous areas leads to the presence of smaller fragments that can rearrange into crystalline structures.^53,65^ Such a mechanism was further confirmed by the presence of two distinct peaks in the melting area, suggesting that two different crystalline populations were present in the materials. The recrystallized areas of the amorphous regions seemed to possess a less perfect structure, therefore melting at a lower temperature (40 °C compared to 50 °C).^58^ The increase in the enthalpy of fusion could also be related to the decrease in the rate of reduction of the molecular weight over time, as the new crystalline regions can act as a barrier to water diffusion and hence decrease the rate of hydrolysis.^64^ Finally, a slight decrease in the pH of the solution was detected from 7.4 to 7.2 over the degradation tests. This result is encouraging for the application of such devices for tissue engineering, as polyesters like PLLA or PLGA are usually associated with a reduction of the local pH due to their degradation products, with possible adverse effects such as the occurrence of a late inflammation at the implantation site.^66,67^ In the literature, PHAs have been shown to possess less acidic degradation products than the above-mentioned polyesters, making them attractive alternative materials.^68,69^

The compatibility of the 3D printed P(3HO-*co*-3HD-*co*-3HDD) scaffolds was evaluated by preliminary *in vitro* studies using MC3T3-E1 cells. The indirect cytotoxicity test confirmed the absence of toxic leachable compounds in the developed constructs. Moreover, the materials were able to support the growth and proliferation of the cells and a continuous and uniform layer of cells was detected after 7 days of incubation. To further investigate the use of these materials in bone regeneration, differentiation studies were conducted using a differentiation medium containing ascorbic acid and β-glycerophosphate to favor MC3T3-E1r differentiation into osteoblasts, and the differentiation was evaluated in terms of ALP activity and calcium deposition (i.e., alizarin red assay). Under such conditions, the cell line undergoes three phases: proliferation, maturation, and mineralization. In the first phase, the cells attach and grow on the surface of the material but are still at an immature stage with low levels of ALP and calcium deposition.^70^ In this study, this early phase was detected on both the scaffolds and the control (i.e., tissue culture plastic) as both markers were low after 7 days of incubation of the MC3T3-E1 cells. The maturation phase usually starts after 10 days of incubation and is characterized by a peak of ALP production, which then remains constant or even decreases during the final phase. The final stage typically begins after 16 days and is characterized by the deposition of inorganic calcium in the extracellular matrix, with the highest deposition at the end of incubation.^71,72^ The cells cultured on TCP showed this typical behavior, with the maximum ALP activity observed after 14 days of incubation followed by the highest increment of calcium deposition after 21 days. For the 3D printed scaffolds, the ALP peak increased over time, but the highest value was detected at 21 days, indicating a possible slower maturation of the cells on such constructs compared to TCP. Nevertheless, the maximum ALP activity was comparable to that of TCP, and a high level of mineralization was achieved after 21 days, without significant differences with the TCP, indicating that the 3D printed P(3HO-*co*-3HD-*co*-3HDD) structures can support the differentiation of the cells *in vitro*. To obtain a better understanding of the influence of the 3D structure on the differentiation of the MC3T3-E1 cells compared to 2D systems, the same material should be evaluated in future studies, using P(3HO-*co*-3HD-*co*-3HDD) solvent cast films, eliminating possible differences due to the intrinsic material properties.^73,74^ Finally, an osteoinductive behavior for such materials was not detected in this study, which was expected as PHAs are not bioactive polyesters.

In the second part of the work, the addition of antimicrobial properties to the 3D printed P(3HO-3HD-3HDD) scaffolds was explored. Commercially available antimicrobial strategies currently applied in the clinic rely heavily on the use of antibiotics, through either systemic administration or drug-loaded spacers or grafts. For these reasons, in the last few years, researchers have been focusing on developing antibiotic-free scaffolds for bone regeneration. In terms of 3D printed polymer-based materials, few studies have been conducted on the production of antibacterial PCL or PLA scaffolds through the incorporation of therapeutic ions (i.e., copper, zinc, or silver) or natural products (i.e., chitosan and poly-ε-lysine).^5,75^ In this work, two strategies were investigated, the development of 3D antibacterial composites and of 3D inherently antibacterial materials using PHAs.

The first strategy to develop 3D PHA-based antibacterial material was based on the use of thioester-containing PHAs to obtain antibacterial materials. Such inherently antibacterial materials could open ways of making constructs possessing long-term activity and durability thanks to the chemical stability of the antibacterial moieties.^76^ 2D 80:20 blend films of P(3HO-*co*-3HD-*co*-3HDD) and PHACOS showed antimicrobial properties against *S. aureus* 6538P and compatibility toward MC3T3-E1 cells, while still maintaining good handleability and processability. From a mechanical point of view (tensile tasting data not shown), 2D 80:20 blend films exhibited the typical elastomeric behavior of mcl-PHAs, with an average elastic modulus of 3 MPa, ultimate tensile strength of 3 MPa and an elongation at break of around 400%. For these reasons, this combination was chosen as the starting material to produce 3D printed scaffolds. Limited work has been reported in the literature regarding the application of PHACOS as materials in the medical field. Dinjaski et al. performed preliminary biocompatibility studies of the material applied in the form of coatings on PET disks using BALB 3T3 fibroblasts, showing that the polymer does not show any detrimental effect against these mammalian cell lines. The materials were also implanted *in vivo* subcutaneously in mice, inducing low inflammation, and still possessing antibacterial activity.^31^ Finally, PHACOS were blended with bacterial cellulose to produce inherently antibacterial hydrogels for wound healing applications.^32^ In our study, the possibility of applying PHACOS in bone tissue engineering applications was evaluated for the first time. Moreover, successful printing of the blend composite was demonstrated for the first time, showing the potential of broader utilization of such materials. Chemical characterization of the scaffold was conducted through FT-IR analysis, which confirmed the presence of the thioester groups on the surface of the produced scaffolds. The 3D printed blend samples showed the presence of an additional peak at 1690 cm^–1^ related to the stretching of the carbonyl group of the thioester bond.^77^ The presence of sulfur was also confirmed through EDX analysis. The antibacterial properties of the 3D printed blend scaffolds were investigated using ISO 22196 against *S. aureus* 6538P. Reduction of the bacterial cell count was detected, showing that the 3D materials still preserved the antibacterial properties of the 2D samples used as starting materials. Furthermore, preliminary *in vitro* compatibility studies were performed by seeding MC3T3-E1 cells on the scaffolds for 1, 3, and 7 days. The materials showed good cell viability for all of the time points evaluated, confirming their potential for bone regeneration.

For the second strategy, 3D composite scaffolds were investigated exploiting a previously developed material based on the combination of P(3HO-*co*-3HD-*co*-3HDD) as the bulk material and Se–Sr–HA as the filler.^33^ Composite materials have been widely investigated for bone regeneration as they highly mimic the intrinsic composition of the tissue: the organic phase (the extracellular matrix containing mainly collagen fibrils), the inorganic phase (mainly hydroxyapatite), and water. Hydroxyapatite (Ca_10_(PO_4_)_6_(OH)_2_) is the main ceramic material used in the fabrication of composite scaffolds for bone regeneration as its chemical and crystal structure closely resembles that of natural hydroxyapatite.^15,73^ This material has shown good biocompatibility, nontoxicity, osteoconductive properties, and osteointegration. As shown in our previous work,^33^ HA doping with selenium and strontium imparts bactericidal properties to the ceramic against both Gram-positive and Gram-negative bacteria. In this work, successful printing of scaffolds containing 10 wt % Se–Sr–HA was achieved. Chemical analysis of the developed materials through ATR-FT-IR confirmed the presence of hydroxyapatite in the scaffolds, showing characteristic peaks of phosphate groups typical of HA. EDX analysis of the 3D composite scaffolds further confirmed the presence of Se–Sr–HA, as all the elements present in this filler could be identified (i.e., selenium, strontium, calcium, and phosphorus). To the best of our knowledge, this is the first attempt at investigating the possibility of developing mcl-PHA-based 3D composite constructs using 3D melt extrusion. A similar processing technique was employed by Yang et al. to produce P(3HB-*co*-3HHx) scaffolds which were then coated with bioactive glasses to improve the osteogenic properties of the material surface.^24^ In another study, the same PHA was used as an additive material to produce 3D constructs based on bioactive glasses through a solution-based 3D printing system using a mixture of the polyester and the particles in a chloroform and dimethyl sulfoxide solution. The role of the polymer was to act as a polymer binder to improve material toughness and reduction of its brittleness.^23^ In this work, P(3HO-*co*-3HD-*co*-HDD) was used as the main material to produce bone scaffolds for potential nonload-bearing applications, where the elastomeric nature of the polyester would allow the development of surgically friendly, flexible, and easy-to-shape constructs able to adapt to the injured or diseased site.^78,79^ Indeed, the mechanical properties of P(3HO-*co*-3HD-*co*-3HDD) were already reported in previous studies,^33,34^ showing an elastic modulus of 5 MPa, ultimate tensile strength of 6 MPa and a high elongation at break (above 400%). The introduction of hydroxyapatite as a filler is expected to improve both the mechanical and the biological properties of the scaffolds. The novel Se–Sr–HA was chosen to produce a material with superior biological properties due to the presence of therapeutic metals able to induce bone formation (i.e., strontium ions) and display antimicrobial properties (i.e., selenium ions).^33^ Enhancement of the mechanical properties has already been evidenced in our previous work. The composite materials showed an increased elastic modulus (15 MPa) and a decrease in the elongation at break (ca. 300%), while still possessing a ductile behavior after the incorporation of hydroxyapatite, making them suitable for possible application in bone regeneration even though the mechanical strength of the materials was lower than that of the native tissue.^80,81^ The targeted application for such an elastomeric composite material is indeed for cancellous bone filling agents in nonload-bearing applications, where their elastic nature might be an advantage for the development of flexible and easily shaped materials able to adapt to the defected site.^79,82^

The compatibility of the 3D printed scaffolds was evaluated by preliminary *in vitro* studies using MC3T3-E1 cells through both indirect and direct tests. For the indirect cytotoxicity evaluation, the 3D P(3HO-*co*-3HD-*co*-HDD) scaffolds with 10 wt % Se–Sr–HA did not show any toxic effect for the time points evaluated. A direct cytocompatibility experiment was conducted by seeding the cells directly on the surface of the 3D printed composite scaffolds and culturing them for 1, 3, and 7 days. The 3D composite scaffolds showed good cell viability for all the time points evaluated, displaying potential for their use in bone tissue engineering.

The antibacterial properties of the 3D composite materials were investigated using ISO 22196, a standard procedure that allows to evaluate the antimicrobial activity of samples in direct contact with the bacteria. The 3D composite scaffold induced an average of 80% reduction in the bacterial cell count against both *S. aureus* 6538P and *E. coli* 8739. These results confirmed the activity of Se–Sr–HA against both Gram-positive and Gram-negative species, as already shown in a previous work for 2D composite films.^33^ Further investigation of the antimicrobial properties of the selected 3D P(3HO-*co*-3HD-*co*-3HDD) with 10 wt % Se–Sr–HA scaffold was carried out through an indirect antibacterial ion release study. Like the 2D composite films used as the starting material, the 3D composites should display antimicrobial properties by direct contact, through the ions present in the matrix, and by an indirect mechanism, through the ions released in the environment. The samples showed antibacterial activity against both *S. aureus* 6538P and *E. coli* 8739. These results confirmed the capability of the matrix to release ions in the surrounding media. The ions released seem to be prolonged over time, as a reduction in the bacterial cell count was detected with the eluates obtained after 7 days of incubation with the materials. A slightly higher effect was observed against Gram-positive bacteria compared to Gram-negative ones, in agreement with literature studies.^83,84^ The lower activity of the indirect antibacterial test compared to the results of ISO 22196 may be due to the lower concentration of ions released in the media compared to those present on the surface of the materials. Nevertheless, to confirm this hypothesis, further studies should be conducted to quantify the ionic content of the media (e.g., via inductively coupled plasma/optical, ICP emission spectroscopy) and to investigate the release over a longer period.^36^ Finally, the time points selected (i.e., 1, 3, and 7 days) for the indirect release study were the same as the *in vitro* cytotoxicity studies, showing once more the potential of the 3D printed P(3HO-*co*-3HD-*co*-HDD) composites with 10 wt % Se–Sr–HA to inhibit bacterial growth while showing good cell viability of mammalian cells.

## 5 Conclusions and Future Work

In this work, the possibility of developing scaffolds using melt-extrusion 3D printing of medium chain length PHAs for bone regeneration was investigated for the first time. The use of additive manufacturing can expand the applications of PHAs in the medical field as the technique can ultimately allow the development of customized and patient-specific scaffolds that match the shape and dimensions of the tissue to be replaced. The process parameters were successfully optimized to produce well-defined and reproducible 3D P(3HO-*co*-3HD-*co*-3HDD) scaffolds. An *in vitro* degradation study in PBS was conducted to investigate the potential of the printed constructs for long-term applications, like bone regeneration. The samples showed a reduction of the molecular weight but no apparent change in their physical structure during the period investigated. The 3D scaffolds developed showed good cytocompatibility toward both undifferentiated and differentiated MC3T3-E1 cells, showing that the material can be used as a suitable substrate for bone regeneration.

Moreover, the possibility of incorporating antibacterial features in the 3D printed P(3HO-*co*-3HD-*co*-3HDD) was investigated via two strategies to develop novel 3D antibacterial constructs based on PHAs for bone regeneration. For the first strategy, inherently antibacterial scaffolds were produced by the melt 3D printing of 80:20 blend films of P(3HO-*co*-3HD-*co*-3HDD) and PHACOS. The structures developed showed antibacterial activity against *S. aureus* 6538P by direct contact using the ISO 22196. The material exhibited good cytocompatibility toward MC3T3-E1 cells, showing potential for bone regeneration. The second strategy involved the production of novel 3D composite scaffolds using the 2D films developed in a previous study, obtained by the combination of P(3HO-*co*-3HD-*co*-3HDD) and Se–Sr–HA. The composite materials with 10 wt % Se–Sr–HA as a filler, 3D P(3HO-*co*-3HD-*co*-3HDD)/10 wt % Se–Sr–HA were successfully printed for the first time and showed a dual mechanism of antibacterial activity by direct contact and through the release of active ions at a concentration capable of inhibiting both *E. coli* 8739 and *S. aureus* 6538P.

Future works should investigate the long-lasting effect of the antimicrobial action of the materials against other strains associated with bone infections (e.g., *S. epidermidis*, *Streptococci sp*., *Pseudomonas sp*.) and using other techniques (e.g., live/dead analysis, confocal microscopy). Finally, the two strategies investigated for the development of 3D antibacterial PHA scaffold could be combined in the future, producing osteoinductive, osteoconductive structures possessing long-lasting antibacterial properties by contact and through the ion release.
